# Density Functional Theory Study of the Spin–Orbit Insulating Phase in SnTe Cubic Nanowires: Implications for Topological Electronics

**DOI:** 10.1021/acsanm.4c00506

**Published:** 2024-03-27

**Authors:** Ghulam Hussain, Kinga Warda, Giuseppe Cuono, Carmine Autieri

**Affiliations:** † International Research Centre MagTop, Institute of Physics, Polish Academy of Sciences, Aleja Lotników 32/46, Warsaw PL-02668, Poland; ‡ Institute for Advanced Study, Shenzhen University, Shenzhen 518060, China; § Faculty of Applied Physics and Mathematics, Gdansk University of Technology, Gdańsk 80-233, Poland

**Keywords:** SnTe nanowires, topology, spin−orbit, density functional theory, Majorana

## Abstract

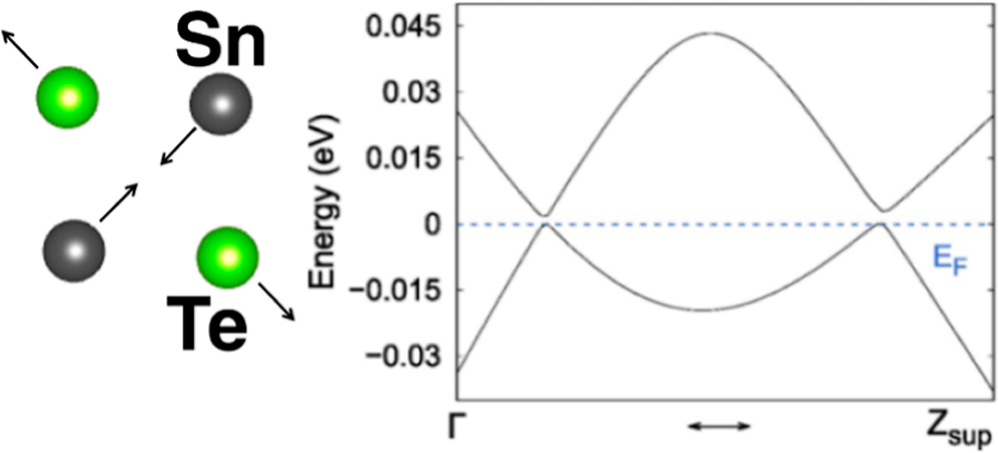

We investigate the electronic, structural, and topological properties of the SnTe and PbTe cubic nanowires using *ab initio* calculations. Using standard and linear-scale density functional theory, we go from the ultrathin limit up to the nanowire thicknesses observed experimentally. Finite-size effects in the ultrathin limit produce an electric quadrupole and associated structural distortions; these distortions increase the band gap, but they get reduced with the size of the nanowires and become less and less relevant. Ultrathin SnTe cubic nanowires are trivial band gap insulators; we demonstrate that by increasing the thickness, there is an electronic transition to a spin–orbit insulating phase due to trivial surface states in the regime of thin nanowires. These trivial surface states with a spin–orbit gap of a few meV appear at the same *k*-point of the topological surface states. Going to the limit of thick nanowires, we should observe the transition to the topological crystalline insulator phase with the presence of two massive surface Dirac fermions hybridized with the persistent trivial surface states. Therefore, we have the copresence of massive Dirac surface states and trivial surface states close to the Fermi level in the same region of the *k*-space. According to our estimation, the cubic SnTe nanowires are trivial insulators below the critical thickness *t*_*c*1_ = 10 nm, and they become spin–orbit insulators between *t*_*c*1_ = 10 nm and *t*_*c*2_ = 17 nm, while they transit to the topological phase above the critical thickness of *t*_*c*2_ = 17 nm. These critical thickness values are in the range of typical experimental thicknesses, making the thickness a relevant parameter for the synthesis of topological cubic nanowires. Pb_1–*x*_Sn_*x*_Te nanowires would have both these critical thicknesses *t*_*c*1_ and *t*_*c*2_ at larger values depending on the doping concentration. We discuss the limitations of density functional theory in the context of topological nanowires and the consequences of our results on topological electronics.

## Introduction

Majorana fermions are intriguing particles that have garnered significant attention in the field of condensed matter physics. Majorana fermions are non-Abelian atoms, meaning their quantum states exhibit intriguing noncommutative properties. They obey non-Abelian statistics, and their topological protection could be crucial for quantum computing and topological quantum information processing. Their potential application in topological quantum computation promises quantum algorithms that are robust against decoherence and errors. Majorana-based qubits are expected to be more robust than other qubit technologies due to their topological protection and are envisioned to play a vital role in quantum error correction protocols.^1,2^

One of the platforms proposed to host Majorana fermions is a topological nanowire (NW) with superconductivity in an applied magnetic field. However, the realization of Majorana’s fermions is challenging as it requires the growth of a NW crystal to cultivate a column of atoms measuring approximately 100 nm in diameter. Subsequently, this system needs to be integrated with a circuit that exhibits the necessary sensitivity to monitor individual electrons as they traverse through it. Moreover, all of this intricate work must be conducted at temperatures merely one-hundredth of a degree above absolute zero and within a magnetic field that is 10,000 times stronger than the earth’s.^3^ NWs made from various materials like semiconductor–superconductor hybrids or topological insulators with proximity-induced superconductivity have been studied to realize Majorana fermions.^4^ Recently, these studies have been extended to cases without inversion symmetry.^5,6^

IV–VI semiconductors have exhibited interesting properties such as thermoelectricity,^7^ ferroelectricity,^8,9^ and superconductivity.^10,11^ In particular, the discovery of the topological crystalline insulator (TCI) phase in SnTe^12−15^ and some of their substitutional alloys such as Pb_1–*x*_Sn_*x*_Te^16^ and Pb_1–*x*_Sn_*x*_Se^17−19^ has aroused tremendous research interest to further study this class of materials. The TCI phase is different from the conventional topological insulating phase; indeed, the linearly dispersing Dirac states on the high-symmetry surfaces of the TCIs are protected by crystal symmetries^20^ and not by time-reversal symmetry. SnTe and its substitutional alloys are TCIs with mirror symmetry, and the presence of the surface states is indicated by a nonzero integer topological invariant named the mirror Chern number.^13,21−25^ Furthermore, it has been shown that SnTe is a helical, higher-order topological insulator.^26−28^ It has been observed that the characteristic properties change by changing the size or dimensions (2D or 3D phase) of materials; namely, the properties will change if we move from bulk^29−31^ to thin films.^32−34^ For instance, SnTe is a trivial insulator at low thickness but becomes topological above some critical thickness^35,36^ and is quite robust against impurity doping.^37^ Recently, the twinning in thin films as a function of the mirror Chern number was studied.^38^ By using scanning tunneling microscopy for Pb_1–*x*_Sn_*x*_Se, we showed that atomically flat terraces in the Se sublattice separated by step edges of various heights are present. Enhancements of the local DOS have been found at odd step edges, and by using tight binding models, spin-polarized flat bands connecting Dirac points have been shown.^39^ The properties of the low-energy states at the surface atomic steps and the behavior of such edge channels under doping were investigated.^11,39−41^

Going to the one-dimensional (1D) case, the TCI SnTe NWs can be even more interesting than the bulk due to a larger contribution from the topological surface states and to the 1D confinement effect.^42^ Methods to produce SnTe NWs by using graphene,^43^ high-yield, and alloy nanoparticles as growth catalysts^9^ or to obtain the smallest possible SnTe NWs by using single-walled carbon nanotubes^44^ were found. TCI NWs are a versatile platform for the confinement and manipulation of Dirac fermions. The Pb_1–*x*_Sn_*x*_Te cubic NWs were also synthesized.^45−47^ Recently, pentagonal NWs with perfect *C*_5_ symmetry have been synthesized in Pb_1–*x*_Sn_*x*_Te. This noncubic crystal structure (impossible in a pure ionic system) arises from the mixture of covalent and ionic bonds. Additionally, the pentagonal phase could be a platform for high-order topology. The results of the investigation on pentagonal NWs will be presented elsewhere. Theoretically, the SnTe NWs were investigated, and it was shown that different topological states can be realized under the application of various magnetic fields;^48^ bulk Majorana modes are present when there is inversion symmetry, while under symmetry-breaking fields at the ends of the NWs, Majorana zero modes appear, while the Majorana bulk modes are gapped.^48^ Ultrathin SnS and SnSe NWs were also studied by means of *ab initio* calculations,^49^ while the NWs of trivial PbTe were also shown to be interesting for the possible presence of Majorana zero modes.^50,51^

The 3D and 2D cases of these rock-salt chalcogenides are widely investigated from first principles, while the 1D structures like NWs are poorly investigated so far due to the huge number of atoms to be considered when we break the boundary conditions along 2 dimensions. As an additional problem within the first principle approach, the description of the IV–VI semiconductors and more generally narrow band semiconductors suffers from the band gap problem that overestimates the topological region of the phase diagram, giving a wrong band ordering. To ease this problem and obtain a description closer to the experiments, we need to go beyond the standard GGA approximation.^23,52−54^ More information on the computational details is reported in the Supporting Information. We find that the thin cubic NWs show robust insulating behavior. We investigate the evolution of the band gap as a function of the thickness for the cubic NWs, and we find the critical thickness at which the system becomes topological. The paper is organized as follows: the next section is devoted to the theoretical investigation of the structural and electronic properties of the cubic NWs, while in the third section, the topology of the NWs is investigated. In the last section, we draw our conclusions.

## Structural and Electronic Properties of the SnTe and PbTe Cubic Nanowires

**Figure 1:**
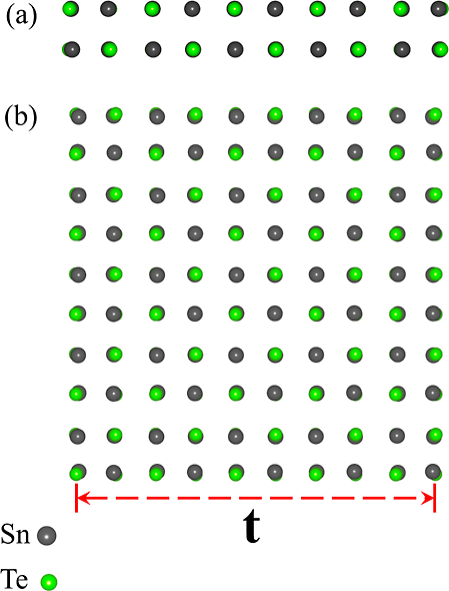
(a) Side view and (b) top view of the crystal structure of the cubic SnTe NWs with an even number of atoms along the *x*- and *y*-direction. We indicate *t* as the thickness of the NW. The unit cell is composed of 2*N* × 2*N* × 2 atoms with periodic boundary conditions along the *z*-axis.

In this section, we investigate the structural and electronic properties of the NWs grown along the [001] direction, focusing on the ultrathin NWs using the VASP code. We investigate unit cells containing 2*N* × 2*N* × 2 NWs, where *N* = 1, 2, 3, ... is the number of atoms, as we can see in Figure 1a. We investigated the SnTe and PbTe NWs with a [001] orientation that present a square section, as represented in Figure 1b. More details are presented in Supporting Information. The (001) surfaces and facets in cubic SnTe are not polar, and the charge present on the inner atoms is almost the same as the surface atoms; therefore, we do not need to introduce dangling bonds. The (001) surface does not show any surface reconstruction, which was instead proposed for the polar surfaces.^55^

**Figure 2:**
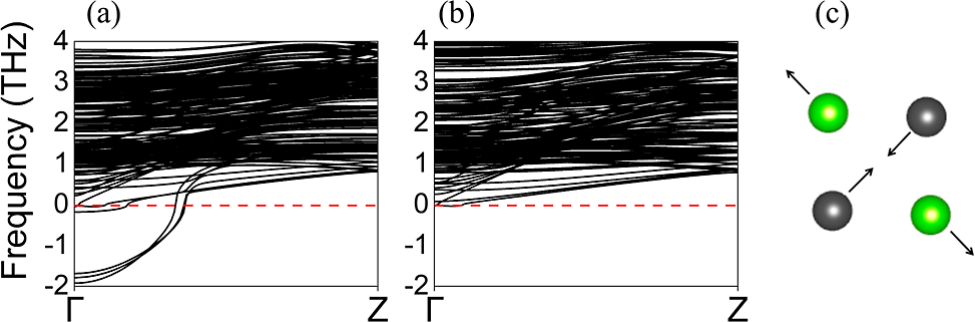
Phonon dispersions for undistorted (a) and distorted (b) cubic NWs. This indicates that the distortions in the cubic systems are important for their dynamic stability. (c) Quadrupolar distortion in the case of the 2 × 2 SnTe NW. The arrows represent the displacements with respect to the ideal NaCl crystal structure. As in Figure 1, the green balls represent the Te atoms, while the dark gray balls represent the Sn atoms.

Ultrathin NWs show distortions from the NaCl phase. The phonon dispersion of the perfectly cubic ultrathin NWs shows in Figure 2a negative frequencies and imaginary phonon modes. Once we include these distortions, all the phonon frequencies of the system become positive, as we can see in Figure 2b. The finite-size effects produce an electric quadrupole with the Sn atoms attracting and the Te atoms repelling each other (Figure 2c). The crystal structure without and with the quadrupolar distortions is shown in the Supporting Information. We have the clear formation of an electric quadruple with positive and negative charges that are slightly different due to the different atomic numbers; this produces nonzero forces on the atoms and displacements along the diagonals. This is very evident in the simple case of the 2 × 2 × 2 NWs, as shown in Figure 2c. The quadrupolar distortions slightly affect the electronic and structural properties at low thicknesses. When we increase the thickness, these quadrupolar distortions are still present on the facets of the NWs while being reduced in the inner parts of the NW. The quadrupolar distortions do not break the inversion, mirror, or nonsymmorphic symmetries.

**Figure 3:**
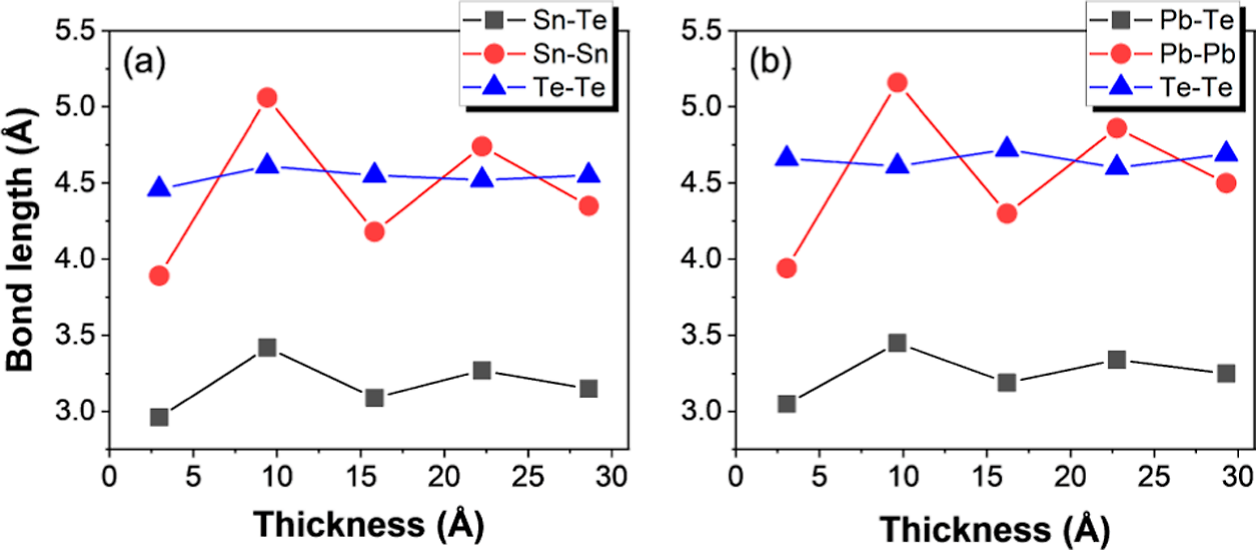
Bond lengths for [001]-oriented cubic NWs as a function of thickness for (a) SnTe and (b) PbTe cubic NWs, respectively. The anion–cation distance is the first-neighbor distance, while the anion–anion and cation–cation distances are the second-neighbor distances. The second-neighbor distances are larger by a factor  with respect to the first-neighbor distances. The line is a guide to the data points.

In Figure 3a,b, we show the bond lengths as a function of the thickness for both the SnTe and PbTe cubic NWs oriented along the [001] direction. We can see that the bond lengths become closer to the theoretical values without relaxation when we go to larger thicknesses. Therefore, these quadrupolar distortions are less and less relevant for thicker NWs, and they will not be considered in the study of the topology. Also, experimentally, it was reported that polar distortions in thin films are more relevant at low thicknesses.^56^

**Figure 4:**
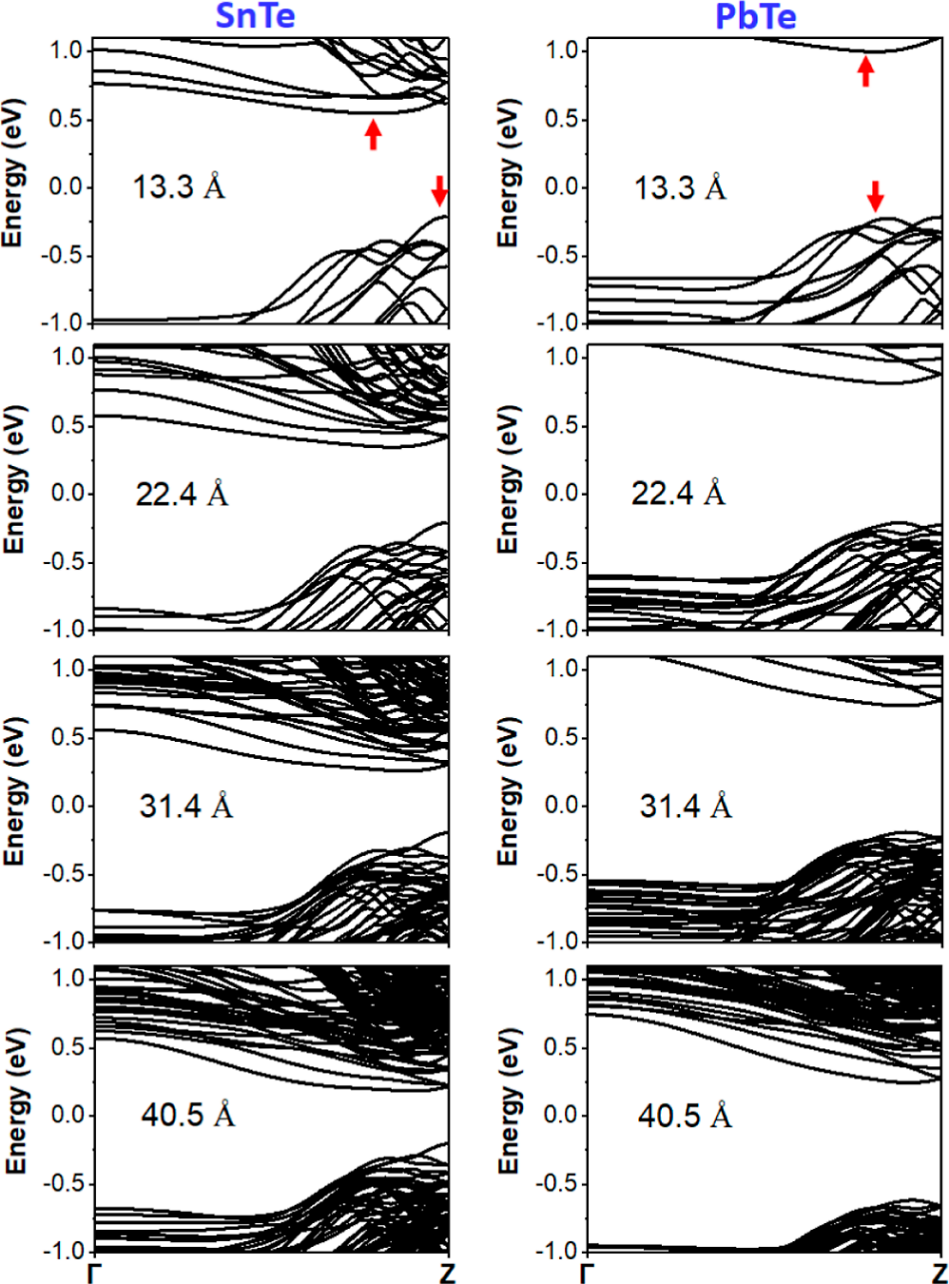
Thickness-dependent electronic band structures of SnTe (left panel) and PbTe (right panel) cubic NWs grown along [001] without SOC. All the cubic NWs reveal trivial insulating behavior. The Fermi level (*E*_F_) is set to zero. The arrows indicate the valence band maximum and conduction band minimum. The conduction bands become closer and closer to the Fermi level as the thickness increases, which decreases the band gap. The thickness is shown in Å, and the conversion to the size is reported in Table 1. Calculations were performed using the VASP code.

Moving to the electronic properties, as all the structures are 1D, we do not need to provide the density of states (DOS) since the band structure gives information about the entire Brillouin zone. We first present the electronic band structures without spin–orbit coupling (SOC) of the SnTe and PbTe cubic NWs oriented along the [001] direction in Figure 4. All these NWs with a size of 10 × 10 or smaller exhibit the trivial nature of insulators, thereby revealing explicit band gaps. Going to the SOC calculations, Table 1 presents the quantitative values of band gaps for SnTe and PbTe cubic NWs, with the band gap decreasing as a function of the thicknesses. As for the bulk systems, we also show that for NWs, the trivial band gap of PbTe is larger than the gap of SnTe. The alloying of Pb_1–*x*_Sn_*x*_Te would likely produce an intermediate gap between PbTe and SnTe. Moreover, the nature of the band gap is different for the two compounds, *i.e.*, SnTe cubic NWs show indirect band gaps, whereas direct band gaps appear for PbTe cubic NWs.

**Table 1:** Size of the NW, the Thicknesses *t* (nm) Equal for Both SnTe and PbTe NWs, and the Total Band Gaps (eV) from SOC Calculations for SnTe and PbTe Cubic NWs after Structural Relaxation Using the VASP Code

size	*t*	gap SnTe	gap PbTe
4 × 4	1.33	0.762	1.215
6 × 6	2.24	0.560	1.028
8 × 8	3.14	0.453	0.929
10 × 10	4.05	0.388	0.865

## Phase Diagram of the Cubic SnTe NWs as a Function of the Thickness

The topological crystalline phase and the associated mirror Chern number are protected by mirror symmetry with respect to the (110) plane. The bulk SnTe has 6 band inversion points in the Brillouin zone, and the 2D topological SnTe with (001) surface orientation hosts 4 band inversion points at nontime-reversal-invariant momenta.^57^ When the 2D Brillouin zone is projected on the 1D Brillouin zone of a NW with (100) and (010) facets, two Dirac points are projected in Γ and become gapped due to their hybridization. The other 2 surface Dirac points of the (001) topological NWs rise close to +*Z* and −*Z*. Due to the large number of electronic bands, we cannot perform the wannierization to have an eventual topological characterization as it was done for the quaternary alloy bulk.^23^ However, we can study the band gap closure to search for the surface Dirac cones in the band structure of the NWs.

**Figure 5:**
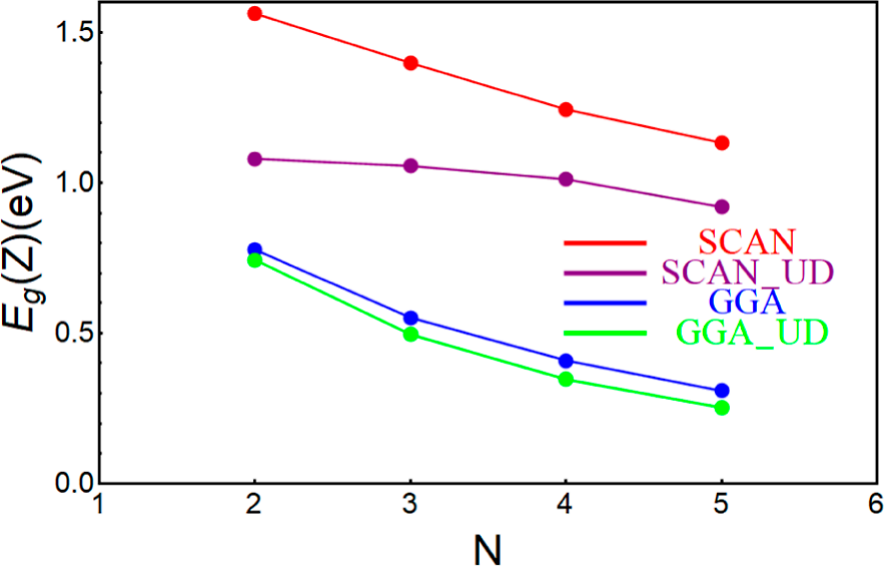
Band gap of SnTe at the *Z* point as a function of NW’s thickness for GGA (blue) and SCAN (red). Undistorted (UD) NW band gaps are reported for GGA and SCAN in green and purple, respectively. The solid lines are a guide for the eyes. The in-plane number of atoms is 2*N* × 2*N*. Calculations were performed using the VASP code.

Here, we investigate the band gap of the SnTe cubic NWs as a function of the thickness using GGA and SCAN metaGGA exchange functional correlations and with the inclusion of the SOC within VASP (Figure 5). We study the band gap at the high-symmetry points that are the relevant points for the topological properties. As the thickness of the NWs increases, the band gap decreases for both the functionals considered. In the metaGGA approximation, the gap is larger, and the electronic phases of the NWs are more far from the topological phase. For all the values of the thicknesses investigated within VASP, the system is trivial in both approximations. To check the effects of the structural relaxation on the band gap, we also calculated the band gap for the undistorted system, namely, without performing structural relaxation. Although the gap without structural distortions is lower in energy than the gap of the distorted structure, in all cases analyzed, the system is trivial.

**Figure 6:**
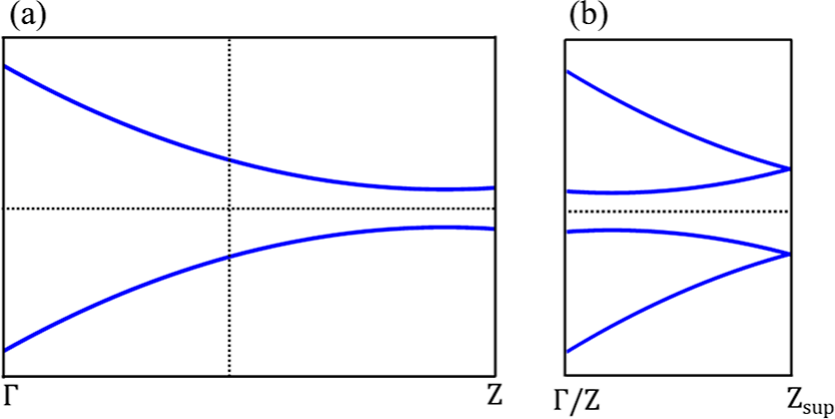
(a) Schematic band structure of 1D TCI with the surface Dirac point close to the *Z* point with . The vertical dashed line represents half of the Brillouin zone. (b) Schematic band structure of 1D TCI with the folding of the *k*-path (due to the doubling of the real space unit cell). The *Z* point is folded in Γ, and the surface Dirac point moves close to Γ. The new Brillouin zone ranges from Γ to the *Z* of the supercell () that is half of the initial *Z*. The horizontal dashed line is the Fermi level set to zero.

In order to increase the thickness of the SnTe NWs, we need to study larger supercells, and we approach this using the linear scaling ONETEP code.^58^ Due to technical reasons, within the ONETEP code, we are forced to double the structure along the *z*-axis. This doubling in the real space produced a halving of the first Brillouin zone in the *k*-space. The high-symmetry point *Z* [see Figure 6a] is folded at the Γ point; therefore, the band closing present close to *Z* moves close to Γ. The Brillouin zone of the supercell extends up to the *Z* of the supercell (*Z*_sup_). This scheme is shown in Figure 6b. Therefore, the band gap at point Γ within the supercell calculations (ONETEP) must be compared with the band gap at *Z* of the unit cell (VASP). Once we have considered this change of the supercell, the agreement between the VASP and ONETEP results is quite good, confirming the reliability of the computational setup of both codes.

**Figure 7:**
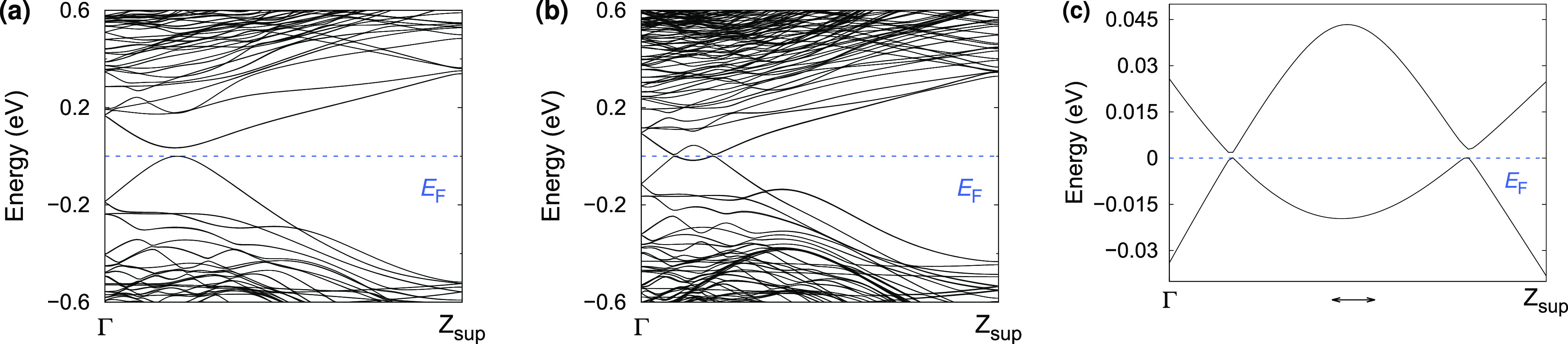
Band structure of the SnTe NWs with different sizes. (a) Band gap insulating phase for the 10 × 10 × 4 NW, (b) spin–orbit insulator for the 14 × 14 × 4 NW, and (c) magnification of the 14 × 14 × 4 NW. The abscissa axis has a range in reduced coordinates between 0 and 0.25 in panels (a,b), while in panel (c), we have a smaller range between 0.016 and 0.060. The calculations have been performed within ONETEP; the Brillouin zone ranges from Γ to *Z*_sup_. The Fermi level is set to zero.

We plot in Figure 7 the whole band structure for the two representative cases, namely, the 10 × 10 × 4 and the 14 × 14 × 4 NWs, and its magnification. Figure 7a shows the band insulator phase that appears in the ultrathin limit for the 10 × 10 × 4 NW or thinner. Upon increasing the size, the cubic SnTe NWs show some trivial surface states that fill the gap, as shown in 7b. The spin–orbit opens a few meV gap between the valence and conduction bands, creating an insulator, as shown in Figure 7c. Since the gap was not created at a high-symmetry point, this is not a topological insulating phase but a trivial spin–orbit insulating phase. This tiny gap could be related to the presence of nonsymmorphic symmetries.^40^ Considering the folding, the position of the trivial surface state is around the *k*-point  in the unfolded Brillouin zone. When the band gap at the high-symmetry point reaches around 0.25 eV, we have the first transition from a band gap insulator to a spin–orbit insulator. These trivial surface states appear on a wider range of thicknesses with respect to the topological surface states; therefore, they are quite robust even if not topologically protected. These trivial surface states lie at the same energy and *k*-point where the topological surface states should form the Dirac point. Since these trivial surface states would persist in the topological phase, we would have a copresence of trivial and topological surface states at around . The presence of these trivial bands would increase the number of carriers in SnTe and, in general, in 1D Pb_1–*x*_Sn_*x*_Te systems. These electronic and topological properties are strongly dependent on the NW’s thickness, as experimentally confirmed.^59^

The *ab initio* calculations will be closer to the experimental materials with respect to the model Hamiltonian, where the same SOC term and the same hopping parameters are used for Sn and Te atoms. Within *ab initio* calculations, we find these additional trivial surface states that are also present in the model Hamiltonian calculations;^48^ however, the spin–orbit gap is much smaller with density functional theory (DFT). Therefore, the presence of trivial surface bands at the Fermi level beyond the surface Dirac bands is not an artifact of the application of the 3D model Hamiltonian to the 1D system but a real property of the IV–VI NWs. These trivial surface states hybridize with the surface Dirac points since they are at the same energy and the same *k*-point; this hybridization produces massive Dirac points.^24^ To have a closed Dirac point, we need to go to very high thicknesses^26^ to avoid hybridization between the facets. Some elements of high-order topology were reported in model Hamiltonian papers even without breaking the mirror symmetry; indeed, the hinge states in cubic NWs are present for large thicknesses at the point *Z*(48) or very close to *Z*.^60^

**Figure 8:**
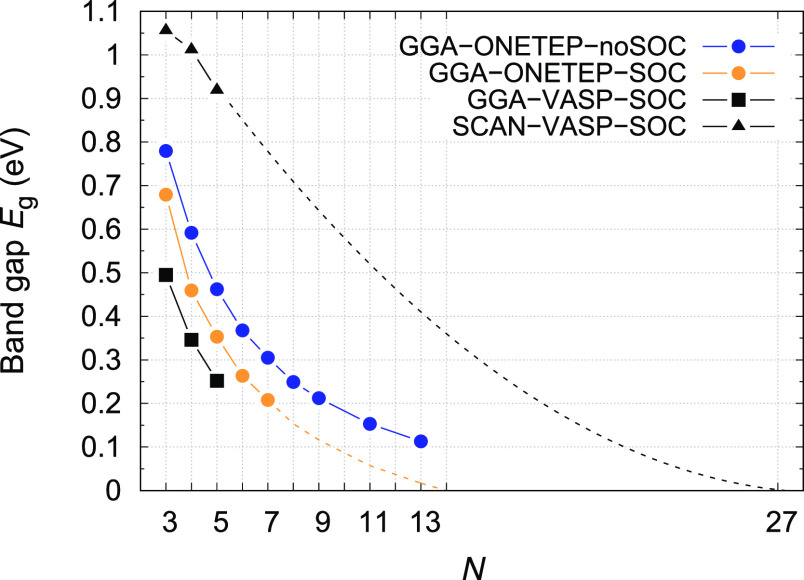
Band gap of SnTe at the Γ point as a function of NW’s thickness without and with SOC. The in-plane number of atoms is 2*N* × 2*N*. Calculations were performed using the ONETEP code with four atoms in the unit cell along the *c*-axis. The gap at the Γ point with four atoms along the *c*-axis is equivalent to the gap at the *Z*-point reported in Figure 5. Blue points represent the gap without SOC, while yellow points represent the gap with SOC. The square points represent the band gap calculated with VASP without distortions. The solid lines are a guide for the eyes. We extrapolate the band gap going to zero from a parabolic fitting of the ONETEP data with SOC. The extrapolation of the SCAN data was performed with a linear fitting for gaps above 0.4 eV and a parabolic trend for the gap below 0.4 eV as from the band gap without SOC.

In Figure 8, we report the band gap of SnTe at the Γ point as a function of the film thickness without and with SOC by using the supercell with 4 atoms along the *c*-axis and the ONETEP code. We compare it with the band gap calculated with VASP within GGA and SCAN. In all cases, the gaps go to zero, increasing the number of atoms and, therefore, the number of hoppings and the total bandwidth. Examining the ONETEP calculations with and without SOC, we observe that the band gap becomes smaller with SOC due to the band splitting in the valence band and conduction band. Upon increasing *N*, the reduction of the gap becomes lower and lower; this is due to the increasing hybridization at the Γ point between the topmost valence band and the lowest conduction band. The quantitative difference between VASP and ONETEP is attributed to different implementations and approximations of the two DFT codes. The SCAN results are expected to be the ones closer to the experimental results; we extrapolate the SCAN results in order to obtain realistic values for the topological transition thicknesses. This extrapolation is quite rough due to the technical limitations of the SCAN method.

**Figure 9:**
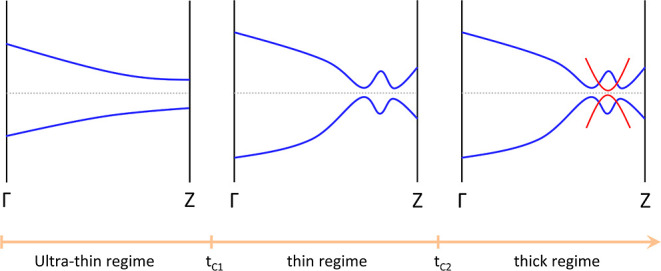
Phase diagram as a function of the thickness *t* for cubic Pb_1–*x*_Sn_*x*_Te NWs. The different phases are separated by the critical thickness values *t*_*c*1_ and *t*_*c*2_. The blue bands are trivial, while the surface massive Dirac fermions are plotted in red. We have a copresence of trivial and topological surface states at around . The horizontal dashed lines represent the Fermi level. Extrapolating the data from SCAN calculations, realistic critical values for pure cubic SnTe NWs are *t*_*c*1_ = 10 nm and *t*_*c*2_ = 17 nm. We expect the same phase diagram for Pb_1–*x*_Sn_*x*_Te NWs but with larger values of *t*_*c*1_ and *t*_*c*2_ depending on the doping concentration.

Within the ONETEP calculations, the first transition from the band insulator to the SOC insulator happens at *N* = 6 when the gap at Γ goes below 0.3 eV, while the topological transition happens at *N* = 14 when the band gap of the system with SOC goes to zero according to the extrapolation in Figure 8. The critical thickness of the NWs is given by *t* = (2*N* – 1)*d*_SnTe_, where *d*_SnTe_ is the distance between Sn and Te, which is half of the lattice constant. From this formula, we calculate the critical values, and we obtain *t*_*c*1_^GGA^ = 3.5 nm and *t*_*c*2_^GGA^ = 8.5 nm for the GGA ONETEP calculations. However, we know that a more realistic value for the topological transition would be within the metaGGCA SCAN. We extrapolate from the SCAN data the behavior up to 0.4 eV; below 0.4 eV, we assume that the reduction of the gap is slowed down by the hybridization between conduction and valence bands, as we can see in the ONETEP results without SOC. Therefore, we assume that behavior for the gap. From our assumptions, we get the first transition at *N* = 16 and the second transition at *N* = 27. Since SCAN is also good for surfaces,^61^ we start from the SCAN results and extrapolate the data, adding the behavior of the NOSOC that we were able to send to zero. We obtain that the system reaches a gap of *ca.* 0.25 eV for *N* = 16, which corresponds to *t*_*c*1_ = 10 nm, while the critical value of the topological transition is *N* = 27, which is equivalent to *t*_*c*2_ = 17 nm. A phase diagram of the three regimes: ultrathin, thin, and thick NWs with their schematic electronic properties is reported in Figure 9. The trivial surface states appear in a wider range than the topological surface states; SnTe NWs with thicknesses of 17 nm or below were synthesized;^43^ therefore, we conclude that these NWs are not topological but are in the SOC insulating phase.

## Conclusions

We have performed high-performance calculations within DFT and linear-scaling DFT. Within the linear scaling DFT, the band gap is larger. Additionally, the band gap does not go linearly as a function of the thickness of the NWs. This makes it not possible to reach the topological phase within the present DFT codes. However, we obtained a thickness dependence of the gap. To improve the present results within DFT accuracy, one of the few possible options is to move to the density functional-based tight binding method, which is based on a second-order expansion of the Kohn–Sham total energy with respect to charge density fluctuations.^62^ In order to investigate Majorana fermions, the SnTe NWs should host superconductivity beyond the topological properties demonstrated in the previous section. The superconductivity can be realized using the proximity effect^63^ by doping^64^ or interfacing SnTe with PbTe.^65,66^ The possibility of ferroelectricity of the cubic phase is addressed in a recent experimental paper,^9^ and further studies are necessary on ferroelectricity and its interplay with the topology. In SnTe, the ferroelectricity produces the Rashba effect because the inversion symmetry is broken; in PbTe, we have strong SOC; then in Pb_1–*x*_Sn_*x*_Te, due to the combination of lack of inversion symmetry and strong SOC, we can have the ferroelectric Rashba topological system.^67,68^

Regarding the experimental surface orientation of the facets, the first NWs grown in 2013 were far from being perfect. Nowadays, experimental NWs can be grown free of defects,^43^ and it is possible to select the desired (001) facet or other facets.^69^ Therefore, our numerical simulations considering defect-free cubic NWs with the (001) orientation of the facet can give results applicable to the experimental conditions. Regarding other facets, such as (110) or (111), the topological aspects are strongly affected by the surface orientation. For instance, while the (001) surface of the bulk SnTe is a crystalline topological insulator, differently, the (111) surface of the bulk SnTe shows two inequivalent Dirac cones at Γ and *M* points.^57^ However, the band gap reduction is a property that is weakly dependent on the surface orientation. Therefore, the trend for the band gap (reduction suppressed in a nonlinear behavior as the number of atoms *N* increases) found in this paper would be the same for other orientations, while the topological aspects would differ. Our numerical approach is not limited to the (001) orientation and could be repeated for different facet orientations, such as (110) or (111) facets.

In conclusion, we have shown using DFT that the thin cubic SnTe and PbTe NWs are trivial insulators in the ultrathin limit. A quadrupolar distortion relevant to the facets of the NWs slightly increases the band gap. At the same thicknesses, the PbTe NWs have a larger trivial gap than that of SnTe NWs. The cubic SnTe NWs undergo an electronic transition from the band gap insulator to a spin–orbit insulator at the thickness *t*_*c*1_ = 10 nm with the phase being still topologically trivial. Upon increasing the thickness again, the cubic SnTe NWs become TCIs for a thickness larger than *t*_*c*1_ = 17 nm. In the topological phase, the SnTe NWs host two massive Dirac surface states at nontime-reversal invariant momenta close to the *Z* point, hybridizing with trivial surface states. The SnTe cubic NWs experimentally reported by different authors with thicknesses ranging between 10 and 200 nm^9,43,70^ are mainly topological systems except the thin NWs with a thickness smaller than *t*_*c*2_ = 17 nm. The IV–VI NWs are further from the topological phase with respect to the IV–VI bulk systems; therefore, the Sn concentration necessary to activate the topological transition in Pb_1–*x*_Sn_*x*_Te will be higher than the bulk values and dependent on the thickness of the NWs. Moving to Pb_1–*x*_Sn_*x*_Te NWs, the values of *t*_*c*1_ and *t*_*c*2_ could sensibly increase, especially close to the level of doping that induces the topological transition in the bulk. Since experimental SnTe cubic NWs are topological and could host superconductivity as well, we conclude that these systems are a suitable platform for the investigation of Majorana fermions.

## Supporting Information Available

Computational details and quadrupolar distortions (PDF)
