# Supplementary material for: Density Functional Theory Study of the Spin–Orbit Insulating Phase in SnTe Cubic Nanowires: Implications for Topological Electronics
Source: ACS Appl Nano Mater. 2024 Mar 27;7(7):8044–52. doi: 10.1021/acsanm.4c00506 (PMC11019662; doi:10.1021/acsanm.4c00506)
Supplement: Supplementary file 1 — an4c00506_si_001.pdf [file an4c00506_si_001.pdf]

Density Functional Theory Study of Spin-Orbit Insulating Phase in SnTe Cubic Nanowires: Implications for Topological Electronics

Ghulam Hussain,[*,†,‡] Kinga Warda,[†,¶] Giuseppe Cuono,[*,†] and Carmine Autieri[*,†]

†*International Research Centre MagTop, Institute of Physics, Polish Academy of Sciences, Aleja Lotników 32/46, PL-02668 Warsaw, Poland*

‡*Institute for Advanced Study, Shenzhen University, Shenzhen 518060, China*

¶*Faculty of Applied Physics and Mathematics, Gdansk University of Technology, Gdańsk 80-233, Poland*

E-mail: ghussain@magtop.ifpan.edu.pl; gcuono@magtop.ifpan.edu.pl; autieri@magtop.ifpan.edu.pl

Abstract

We investigate the electronic, structural and topological properties of the SnTe and PbTe cubic nanowires using *ab initio* calculations. Using standard and linear-scale density functional theory, we go from the ultrathin limit up to the nanowires thicknesses observed experimentally. Finite-size effects in the ultra-thin limit produce an electric quadrupole and associated structural distortions, these distortions increase the band gap but they get reduced with the size of the nanowires and become less and less relevant. Ultrathin SnTe cubic nanowires are trivial band gap insulators, we

demonstrate that by increasing the thickness there is an electronic transition to a spin-orbit insulating phase due to trivial surface states in the regime of thin nanowires. These trivial surface states with a spin-orbit gap of a few meV appear at the same k-point of the topological surface states. Going to the limit of thick nanowires, we should observe the transition to the topological crystalline insulating phase with the presence of two massive surface Dirac fermions hybridized with the persisting trivial surface states. Therefore, we have the co-presence of massive Dirac surface states and trivial surface states close to the Fermi level in the same region of the k-space. According to our estimation, the cubic SnTe nanowires are trivial insulators below the critical thickness t_{c1}=10 nm, and they become spin-orbit insulators between t_{c1}=10 nm and t_{c2}=17 nm, while they transit to the topological phase above the critical thickness of t_{c2}=17 nm. These critical thickness values are in the range of the typical experimental thicknesses, making the thickness a relevant parameter for the synthesis of topological cubic nanowires. $Pb_{1-x}Sn_xTe$ nanowires would have both these critical thicknesses t_{c1} and t_{c2} at larger values depending on the doping concentration. We discuss the limitation of the density functional theory in the context of topological nanowires and the consequences of our results on topological electronics.

Keywords

SnTe Nanowires, topology, spin-orbit, density functional theory, Majorana

Supporting Information Available

Computational details

The results were obtained within the framework of the first-principles density functional theory (DFT), the calculations are fully relativistic by considering spin-orbit coupling (SOC) if not mentioned otherwise. The system presents inversion symmetry since we do not assume

ferroelectricity, as a consequence, the systems host the Kramer's degeneracy due to the combination of time-reversal and inversion symmetry. Therefore, all bands reported in this paper are double degenerate and we plot the band structure only along the k-path Γ-Z. The system has a mirror along the (110) and $(1\bar{1}0)$ planes and a nonsymmorphic symmetry respect to the (100) and (010) planes.

While the symmetry of the SnTe bulk is cubic, we investigated the SnTe and PbTe NWs with [001] orientation that present a square section as represented in Fig. 1b) of the main text. We will define these NWs with a square section in the ab plane and infinite along the c-axis as cubic NWs. The facets of the NWs are in the [100] and [010] directions. The lattice constant c is equal to 6.2964 and 6.4277 Å for SnTe and PbTe, respectively, which are the experimental values. These lattice constants are equivalent to twice the anion-cation distance. We limit the study to an even number of atoms along the x- and y-direction to avoid odd-even effects.[1] Indeed, depending on the odd or even number of atoms the system hosts the mirror or nonsymmorphic symmetry.[2] For the VASP calculations, we investigate unit cells containing 2N×2N×2 nanowires, where N=1, 2, 3... is the number of atoms as we can see in Fig. 1a) of the main text. When we use ONETEP, we double the cell along the z-axis studying 2N×2N×4 nanowires with the lattice constant of the superlattice c_{sup}=2c. We define t as the in-plane thicknesses of the nanowires as shown in Fig. 1b) of the main text.

Structural and electronic structure calculations of the thin nanowires were performed with a plane wave basis set and projector augmented wave method using the VASP[3] package. A plane-wave energy cut-off of 250 eV has been used. As an exchange-correlation functional, the generalized gradient approximation (GGA) of Perdrew, Burke, and Ernzerhof (PBE) has been adopted.[4] The description of the IV-VI semiconductors and more in general narrow band semiconductors suffers from the band gap problem that overestimates the topological region of the phase diagram giving a wrong band ordering. To ease this problem and obtain a description closer to the experiments, we need to go beyond the stan-

dard GGA approximation.[5–8] In the case of nanowires, a more realistic band order and band gap can be obtained with the meta-GGA approach named SCAN, namely, the strongly constrained and appropriately normed functional,[9] which is one of the few exchange-correlation functional working well in the presence of SnTe and PbTe surfaces. Both results with GGA and meta-GGA are qualitatively similar but the meta-GGA gives a larger gap that is much closer to the experimental results. We have performed the VASP self-consistent calculations using 1×1×17 for GGA and 1×1×12 k-points centered in Γ for SCAN, while for the band structures, we have used 60 k-points from Γ to Z.

The number of atoms in the system is $N_{ATOMS}=8N^2$, standard DFT codes scale as $O(N^3_{ATOMS})$, therefore, they are scaling as the $O(N^6)$. This poses serious limitations in the study of thick NWs. In order to increase the thickness of cubic SnTe NWs, we need to study a large number of atoms with SOC. This was accomplished through the use of ONETEP,[10,11] a linear-scaling density functional theory (LS-DFT) approach. In the ONETEP code orbitals that are nonorthogonal generalized Wannier functions (NGWFs) are expressed within the basis of periodic sinc (psinc) functions, providing accuracy comparable to the traditional plane-wave DFT software.[12] Simultaneously, the developed approach offers parallel computation, which allows the simulations of thousands of atoms.[13] The ONETEP calculations were performed with the use of PBE functional and PAW pseudopotentials from the JTH library.[14] The investigation of the bandstructure was carried out with the use of spectral function unfolding methodology.[15] The sampling of the reciprocal space consisted of 500 k-points, spaced equally along the z-reciprocal lattice vector. With each of the Sn and Te atoms, there were assigned 9 NGWFs. Considering the study of large systems, for all NGWFs the radius was determined as $R_\phi = 5\ a_0$, allowing the convergence and sufficient accuracy to be maintained. To ensure the requirements of the bandstructure unfolding formalism, the diameter of the NGWFs could not exceed half the length of the simulation cell. Therefore, the size of the nanowire in the axial direction had to be doubled compared to the structure studied within VASP. The basis set consisted of the psinc functions distant from each other

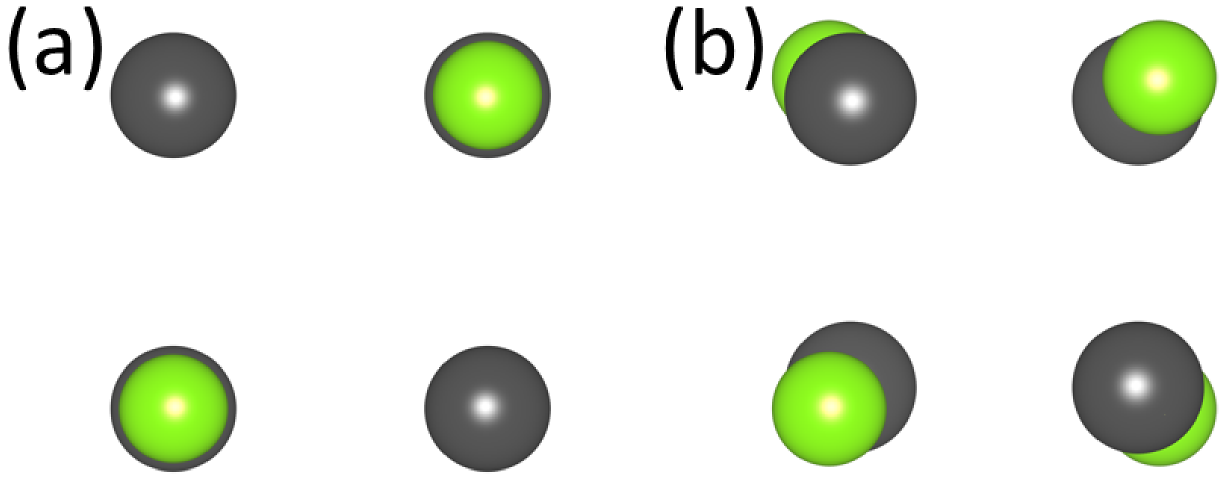

Figure S1: (a) Structure without quadrupolar distortion (b) Structure with quadrupolar distortion in the case of the 2×2 SnTe nanowire. As in Fig. 1 of the main text, the green balls represent the Te atoms, while the dark grey balls represent the Sn atoms.

by $d_{\text{psinc}} = 0.36$ Å, resulting in the density kernel cutoff E_{c} equal 447 eV. The length of the simulation cell in the z-direction was equal to $c_{sup} = 12.5928$ Å, with the imposition of periodic boundary conditions (PBCs). The lengths along the x- and y-directions were selected as multiplies of the d_{psinc} depending on the thickness of the structure, with the vacuum spacing applied. The system investigated consisted of up to 784 atoms for the SOC calculations and 2704 atoms for non-relativistic calculations. The density kernel truncation was not applied. Considering that the ONETEP method is based on localized Wannier functions and is developed for molecules and surfaces, the surface properties calculated within ONETEP would be a reliable benchmark for the calculations obtained with the plane-wave software VASP.

The same numerical approaches were used recently for the study of the $Pb_{1-x}Sn_xTe$ pentagonal nanowires with C_5 symmetry. This non-cubic crystal structure (impossible in a pure ionic system) rises from the mixture of covalent and ionic bonds. Additionally, the pentagonal phase could be a platform for high-order topology. The results of the investigation on pentagonal nanowires will be presented elsewhere.[16]

In Figure 8 of the main text, we have the results with SCAN with spin-orbit which are computationally extremely demanding. We observe that the band gap gets reduced

approximately linearly as a function of N in the region above 0.4 eV for all other cases. When we gap gets reduced the hybridization (bonding-antibonding splitting) slows down the closure of the gap which becomes slower than linear. Below 0.4 eV, we extrapolate the band gap of the spin-orbit calculation with SCAN (black curve) using the behavior of the curve related to the spin-orbit calculations with GGA (yellow curve).

Quadrupolar distortions

We report the crystal structure of SnTe without quadrupolar distortions in Fig. S1(a) and with distortions in Fig. S1(b). Despite the Figure showing a very thin case of the 2 x 2 atoms, it is representative of the general case. The quadrupolar distortion arises due to the charge imbalance between the Sn and Te atoms. There is a positive charge $+|q_1|$ and a negative charge -$|q_2|$. From an electrostatic point of view, this configuration of the charge cannot be a regular square if the two charges are inequivalent. Consequently, these distortions result in minimum total energy for the structures and impart structural stability to the NWs.

References

(1) Chang, K.; Hu, M.; Lin, H.; Liu, J.; Xue, Q.-K.; Chen, X.; Ji, S.-H. Oscillation of Electronic-Band-Gap Size Induced by Crystalline Symmetry Change in Ultrathin PbTe Films. *Phys. Rev. Lett.* **2023**, *131*, 016202.

(2) Brzezicki, W.; Wysokiński, M. M.; Hyart, T. Topological properties of multilayers and surface steps in the SnTe material class. *Phys. Rev. B* **2019**, *100*, 121107.

(3) Kresse, G.; Furthmüller, J. Efficient iterative schemes for ab initio total-energy calculations using a plane-wave basis set. *Physical Review B* **1996**, *54*, 11169.

(4) Perdew, J. P.; Burke, K.; Ernzerhof, M. Generalized gradient approximation made simple. *Physical Review Letters* **1996**, *77*, 3865.

(5) Cuono, G.; Hussain, G.; Fakhredine, A.; Autieri, C. Topological Phase Diagram of Pb1-xSnxSe1-yTey Quaternary Compound. *Acta Physica Polonica A* **2022**, *142*.

(6) Cuono, G.; Sattigeri, R. M.; Autieri, C.; Dietl, T. Ab initio overestimation of the topological region in Eu-based compounds. *Phys. Rev. B* **2023**, *108*, 075150.

(7) Islam, R.; Cuono, G.; Nguyen, M. N.; Noce, C.; Autieri, C. Topological Transition in Pb1-xSnxSe using Meta-GGA Exchange-Correlation Functional. *Acta Physica Polonica A* **2019**, *136*.

(8) Hussain, G.; Cuono, G.; Islam, R.; Trajnerowicz, A.; Jureńczyk, J.; Autieri, C.; Dietl, T. Electronic and optical properties of InAs/InAs0.625Sb0.375 superlattices and their application for far-infrared detectors. *Journal of Physics D: Applied Physics* **2022**, *55*, 495301.

(9) Sun, J.; Ruzsinszky, A.; Perdew, J. P. Strongly Constrained and Appropriately Normed Semilocal Density Functional. *Phys. Rev. Lett.* **2015**, *115*, 036402.

(10) Skylaris, C.-K.; Haynes, P. D.; Mostofi, A. A.; Payne, M. C. Introducing ONETEP: Linear-scaling density functional simulations on parallel computers. *The Journal of Chemical Physics* **2005**, *122*, 084119.

(11) Prentice, J. C. A. et al. The ONETEP linear-scaling density functional theory program. *The Journal of Chemical Physics* **2020**, *152*, 174111.

(12) Skylaris, C.-K.; Haynes, P. D. Achieving plane wave accuracy in linear-scaling density functional theory applied to periodic systems: A case study on crystalline silicon. *The Journal of Chemical Physics* **2007**, *127*, 164712.

(13) Skylaris, C.-K.; Haynes, P. D.; Mostofi, A. A.; Payne, M. C. Recent progress in linear-scaling density functional calculations with plane waves and pseudopotentials: the ONETEP code. *Journal of Physics: Condensed Matter* **2008**, *20*, 064209.

(14) Jollet, F.; Torrent, M.; Holzwarth, N. Generation of Projector Augmented-Wave atomic data: A 71 element validated table in the XML format. *Computer Physics Communications* **2014**, *185*, 1246–1254.

(15) Constantinescu, G. C.; Hine, N. D. M. Energy landscape and band-structure tuning in realistic $MoS_2/MoSe_2$ heterostructures. *Phys. Rev. B* **2015**, *91*, 195416.

(16) Hussain, G.; Cuono, G.; Dziawa, P.; Janaszko, D.; Sadowski, J.; Kret, S.; Kurowska, B.; Polaczynski, J.; Warda, K.; Sattar, S.; Canali, C. M.; Lau, A.; Brzezicki, W.; Story, T.; Autieri, C. Pentagonal nanowires from topological crystalline insulators: a platform for intrinsic core-shell nanowires and higher-order topology. **2024**,
